# Cardiac CT in the Era of Precision Cardiology: From Calcium Scoring to Comprehensive Risk Profiling

**DOI:** 10.3390/jcm15135313

**Published:** 2026-07-07

**Authors:** Gianluigi Napoli, Donatella Tansella, Maria Teresa Savo, Abdulrahman Alsergani, Laura Fusini, Saima Mushtaq, Andrea Baggiano, Fabio Fazzari, Gianluca Pontone, Michele Davide Latorre, Eduardo Urgesi, Maria Cristina Carella, Raffaella Motta, Andrea Igoren Guaricci, Valeria Pergola

**Affiliations:** 1 Cardiology Unit, Villa Verde Clinic, 74121 Taranto, Italy; gianluiginapoli@gmail.com; 2 Cardiology Unit, Mater Dei Hospital, 70125 Bari, Italy; donatellatansella@libero.it; 3 Department of Cardiac, Thoracic and Vascular Sciences and Public Health, University of Padua, 35122 Padua, Italyvaleria.pergola@gmail.com (V.P.); 4 Radiology Unit, King Saud University, Riyadh 11451, Saudi Arabia; alsergani03@gmail.com; 5 Department of Perioperative Cardiology and Cardiovascular Imaging, Centro Cardiologico Monzino, IRCCS, 20138 Milan, Italy; laura.fusini@cardiologicomonzino.it (L.F.); saima.mushtaq@cardiologicomonzino.it (S.M.); andrea.baggiano@cardiologicomonzino.it (A.B.); gianluca.pontone@cardiologicomonzino.it (G.P.); 6 University Cardiology Unit, Interdisciplinary Department of Medicine, Polyclinic University Hospital, 70124 Bari, Italy; latorre.michele.d@gmail.com (M.D.L.); eduardourgesi@gmail.com (E.U.); m.c.carella92@gmail.com (M.C.C.); 7 Radiology Unit, University Hospital of Padova, 35128 Padua, Italy; raffaella.motta@unipd.it

**Keywords:** coronary CT angiography, calcium score, FFR-CT, pFAI, precision cardiology

## Abstract

Cardiac computed tomography (CT) has evolved into a pivotal tool in precision cardiology, enabling comprehensive, non-invasive evaluation of coronary anatomy, plaque composition, vascular function, and inflammation. From calcium scoring to advanced physiological imaging, CT now integrates multiple layers of cardiovascular information within a unified diagnostic framework. Coronary artery calcium (CAC) quantification provides a robust, reproducible measure of atherosclerotic burden and refines risk estimation beyond traditional algorithms, particularly in asymptomatic individuals with an intermediate likelihood. Building upon this anatomical foundation, coronary CT angiography (CCTA) extends evaluation to the anatomical and morphological characterization of coronary artery disease (CAD), identifying both obstructive and non-obstructive plaques with high prognostic accuracy. The addition of CT-derived fractional flow reserve (FFR-CT) and stress perfusion CT (CTP) bridges anatomy and physiology, improving identification of flow-limiting stenoses and guiding revascularization decisions while reducing unnecessary invasive procedures. Beyond luminal assessment, CT-derived biomarkers such as the perivascular fat attenuation index (pFAI) have introduced a new dimension of vascular inflammation imaging, revealing residual risk even in patients without significant stenosis and suggesting novel pathways for individualized therapeutic targeting. Driven by advances in artificial intelligence and photon-counting detector technology, cardiac CT is transitioning from a purely diagnostic modality to an integrative platform for cardiovascular phenotyping. Taken as a whole, this integration of structural, functional, and biological data provides a genuinely holistic view of coronary health. In practical terms, it shifts clinical decision-making from population-based risk models toward precision-guided patient-specific strategies.

## 1. Introduction

Over the past two decades, cardiac computed tomography (CT) has evolved into a pivotal tool in precision cardiology, not merely for anatomical depiction, but as an integrative platform that combines morphological, functional, and biological information [1,2]. Initially limited by spatial and temporal resolution, technological advances (i.e., wide-detector arrays, iterative reconstruction, and artificial intelligence–based post-processing) have transformed cardiac CT into a rapid, reproducible, and non-invasive tool capable of evaluating coronary anatomy, plaque burden, myocardial structure, and even physiological parameters [3].

Among its most transformative applications, the quantification of CAC has redefined cardiovascular prevention. Since the seminal work of Agatston et al. in the early 1990s [4] CAC scoring has become a cornerstone of atherosclerotic cardiovascular disease (ASCVD) risk assessment. The extent of coronary calcification, measurable even in asymptomatic individuals, correlates strongly with future atherosclerotic events [5,6,7]. Large population-based studies, including MESA, have consistently shown that CAC outperforms traditional clinical risk models in predicting coronary heart disease and improves individualized preventive strategies [8,9,10,11]. Both European and American guidelines endorse the integration of CAC into primary prevention algorithms [12,13,14,15]. Recent meta-analyses confirm its value in refining cardiovascular risk estimation, particularly among individuals at intermediate or uncertain risk [16,17]. A zero CAC score confers a robust “warranty period” against cardiovascular events, often allowing clinicians to defer statin therapy in low-risk patients [18,19]. Conversely, high CAC scores (e.g., >1000) identify individuals with event rates comparable to those with established coronary artery disease (CAD), warranting intensive preventive management [20].

Beyond calcium quantification, contrast-enhanced coronary CT angiography (CCTA) has become indispensable for the evaluation of suspected CAD. With excellent negative predictive value, CCTA reliably excludes obstructive disease and prevents unnecessary invasive angiography. Its capacity to depict both luminal stenosis and plaque morphology provides crucial insights into early, non-calcified atherosclerosis. Moreover, its field of view extends beyond the coronary arteries, allowing comprehensive assessment of cardiac chambers, valves, pericardium, and pulmonary vasculature—establishing CCTA as a truly holistic cardiovascular imaging modality [21].

Current guideline recommendations reflect this paradigm shift: the 2021 ESC Guidelines on CVD prevention and 2024 ESC Guidelines on chronic coronary syndromes advocate for the routine integration of cardiac CT in both asymptomatic and symptomatic individuals [12,22]. Landmark trials such as ROBINSCA and SCOT-HEART have demonstrated that CT-based strategies enable earlier diagnosis, more appropriate therapy allocation, and improved outcomes [11,23].

In light of its growing prognostic value, diagnostic accuracy, and expanding indications, cardiac CT is now firmly embedded in contemporary cardiovascular medicine. Whether guiding preventive therapy through CAC, ruling out obstructive disease with CCTA, or identifying high-risk plaque features, cardiac CT stands at the crossroads of innovation and evidence-based care—poised to play a central role in personalized, predictive, and preventive cardiology. Moreover, recent advances in photon-counting CT and deep-learning reconstruction have further improved spatial resolution while reducing radiation dose, enabling comprehensive cardiac evaluation at sub-millisievert exposures.

## 2. Coronary Artery Calcium Score

### 2.1. Technical Principles and Quantification

Coronary artery calcification represents the radiological expression of atherosclerotic plaque mineralization within the coronary arterial wall. Calcified and non-calcified components often coexist within a single lesion, reflecting different stages of plaque evolution. The CAC score quantifies the burden of calcified atherosclerosis, serving as a surrogate marker of total plaque load and an independent predictor of cardiovascular events [24]. Although coronary calcium is traditionally associated with more stable, fibrocalcific lesions, it may also be present in vulnerable plaques, underscoring its role as a cumulative index of atherosclerotic activity rather than a marker of stability alone.

CAC scoring is performed using non-contrast, ECG-gated cardiac CT, typically acquired during late diastole (70–80% of the R–R interval) to minimize motion artifacts and enhance spatial resolution [25]. The standard protocol employs a tube voltage of 120 kV, thin-slice collimation (2.5–3.0 mm), and delivers a low radiation dose (approximately 1 mSv) without contrast material. While CAC can also be visualized on non-ECG-gated chest CT, dedicated ECG-gated acquisitions provide superior accuracy and reproducibility [4].

**Figure 1:**
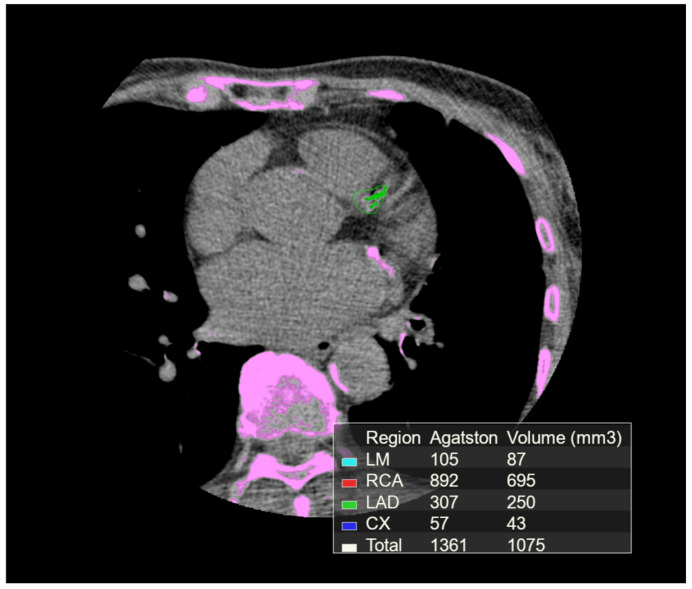
CT-based coronary calcium scoring. Axial non-contrast cardiac CT image showing automated detection and color-coded segmentation of calcified plaques in the coronary arteries, with corresponding Agatston scores and calcium volumes reported for each vessel and for the total coronary calcium burden.

Quantification is based on the Agatston method, which multiplies the calcified plaque area by a density weighting factor derived based on the peak Hounsfield Unit (HU) within each lesion. Individual calcified foci are identified when attenuation exceeds 130 HU and area ≥ 1 mm^2^ [5]. The sum of all lesion scores across coronary arteries yields the total Agatston score, ranging from 0 (no detectable calcification) to values exceeding 1000, which denote extensive, high-risk atherosclerosis [6]. CAC results can also be expressed as age-, sex-, and ethnicity-adjusted percentiles, though absolute values generally offer superior predictive performance for cardiovascular risk and model discrimination [7] (Figure 1).

Beyond total score, emerging evidence has highlighted the prognostic importance of plaque volume and density. In the MESA cohort, higher calcium density was paradoxically associated with lower coronary artery disease risk—particularly at smaller calcium volumes (<130 mm^3^)—suggesting that denser, more compact plaques may represent a more stable phenotype [8]. A large meta-analysis of over 21,000 participants confirmed that higher CAC density independently correlates with reduced cardiovascular risk after adjustment for volume and clinical factors [7,9,26,27,28,29].

Nonetheless, the Agatston score remains the most widely validated and clinically adopted approach. CAC severity shows a graded association with cardiovascular mortality, with scores of 1–100, 101–1000, and >1000 corresponding to hazard ratios for coronary death of 1.27, 3.57, and 6.63, respectively [10]. Thus, the following sections will primarily refer to the Agatston score when discussing CAC-based risk stratification.

### 2.2. CAC Score in Asymptomatic Individuals

The CAC score holds particular clinical value in primary prevention, where it refines cardiovascular risk estimation beyond traditional algorithms. By reclassifying individuals above or below established treatment thresholds, CAC assessment helps tailor therapeutic decisions, especially for patients whose estimated risk lies near statin initiation cut-offs. According to the 2021 ESC Guidelines on cardiovascular disease prevention, CAC scoring may be considered (Class IIb, Level of Evidence B) in individuals with borderline or intermediate risk as defined by SCORE2 or SCORE2-OP [12]. Evidence from the ROBINSCA trial demonstrated that CAC screening significantly reduced the proportion of individuals categorized as intermediate or high risk, suggesting its potential to prevent overtreatment and optimize preventive strategies [11]. Similarly, Pavlović et al. compared CAC-guided reclassification using various U.S. and European risk models (including Pooled Cohort Equations (PCE), PREVENT, and SCORE2) and found that CAC integration improved risk stratification with estimated 10-year numbers needed to treat (NNT) for statin therapy ranging from 11 to 26, consistent across guideline frameworks [14].

Multiple large cohort studies, including MESA, CARDIA, and the Rotterdam Study, have consistently shown that a CAC score of 0 identifies individuals at very low 10-year risk for cardiovascular events [18]. The absence of coronary calcification thus defines a “warranty period” of low event rates and can meaningfully guide clinical decision-making. In particular, CAC = 0 may support the deferral or avoidance of statin therapy in the following scenarios: (1) statin-naïve patients hesitant about initiating therapy, (2) patients with prior statin intolerance considering rechallenge, (3) individuals without major risk factors uncertain about benefit, and (4) intermediate-risk patients for whom treatment may reasonably be deferred.

The CAC Consortium has proposed approximate age thresholds for first-time screening in low-risk populations—around 42 years for men and 58 years for women—with earlier evaluation in diabetic individuals (~37 years in men, ~50 years in women) [17]. Conversely, CAC testing may be less informative or cost-effective in certain groups: (1) young, low-risk adults (<40 in men, <50 in women) without cardiovascular risk factors, (2) very elderly patients (≥80 years), or those with significant comorbidities limiting life expectancy, and (3) patients already receiving statin therapy, given that statins promote plaque stabilization and may increase calcification independent of clinical risk reduction.

Importantly, even minimal (>0) CAC score confers a measurable increase in cardiovascular risk compared with a zero score, while very high CAC levels (≥1000) identify individuals with event rates similar to those observed in secondary prevention cohorts [20]. These findings position CAC scoring as a powerful tool for personalized prevention, enabling clinicians to refine risk communication, optimize therapeutic intensity, and minimize both under- and overtreatment in asymptomatic individuals.

### 2.3. CAC Score in Symptomatic Patients

In patients presenting with chest pain or suspected chronic coronary syndromes (CCS), the CAC score provides valuable diagnostic and prognostic information. The 2024 ESC Guidelines for CCS recommend initial estimation of pre-test probability (PTP) using the Risk Factor–Weighted Clinical Likelihood (RF-CL) model. Among individuals with low clinical likelihood (5–15%), CAC assessment can further refine risk stratification and guide the need for subsequent imaging [15,30].

Absence of CAC (CAC = 0) strongly predicts the absence of obstructive CAD and correlates with a low annual risk of major adverse cardiac events (MACE) [19]. A large meta-analysis involving over 92,000 patients with either stable or acute chest pain demonstrated negative predictive values of 97% and 98%, respectively, for CAC = 0 in excluding obstructive CAD [19]. These data underscore the high rule-out performance of CAC scoring and support its use as an effective “gatekeeper” strategy, helping to avoid unnecessary downstream testing such as CCTA or invasive coronary angiography in appropriately selected low-risk patients. When integrated with clinical models such as the RF-CL, CAC scoring further enhances diagnostic efficiency. Combined assessment allows a greater proportion of patients to be reclassified into the very low-risk category (54% vs. 38% with clinical models alone), substantially reducing the need for advanced imaging and associated healthcare costs (see Table 1).

### 2.4. Calcium Score in the Treatment of Aortic Valve Stenosis

The advent of multiphase cardiac CT has revolutionized the pre-procedural evaluation of severe aortic stenosis and the planning of transcatheter aortic valve implantation (TAVI) by providing detailed three-dimensional anatomical visualization of the aortic root complex, valve leaflets, coronary ostia, and vascular access pathways. These measurements are essential for selecting the appropriate prosthesis size, anticipating the risk of paravalvular leak, guiding C-arm angulation in the catheterization laboratory, and defining the optimal vascular access route and delivery system trajectory (e.g., femoral, subclavian, trans-axillary). In addition, CT enables quantitative assessment of valvular calcification through the aortic valve calcium score (AVCS), a flow-independent metric that supports both diagnostic clarification and procedural planning. AVCS is derived from non-contrast, ECG-gated CT acquisitions and expressed in Agatston units (AU). According to the 2025 ESC/EACTS guidelines, an AVCS of ≥1600 AU in women and ≥2000 AU in men indicates severe aortic stenosis, whereas values below 800 AU in women and 1600 AU in men make severe stenosis unlikely [31].

AVCS is particularly valuable in patients with low-flow, low-gradient (LFLG) aortic stenosis, where echocardiographic parameters may underestimate disease severity. In this context, AVCS helps distinguish true severe from pseudo-severe stenosis, thereby guiding decisions regarding valve intervention. Importantly, because AVCS reflects the anatomical burden of calcification independently of hemodynamic conditions, it provides a reliable surrogate for valvular obstruction and complements other imaging modalities in comprehensive pre-TAVI assessment.

## 3. The Role of Coronary CT Angiography in the Evaluation of Coronary Arteries

**Figure 2:**
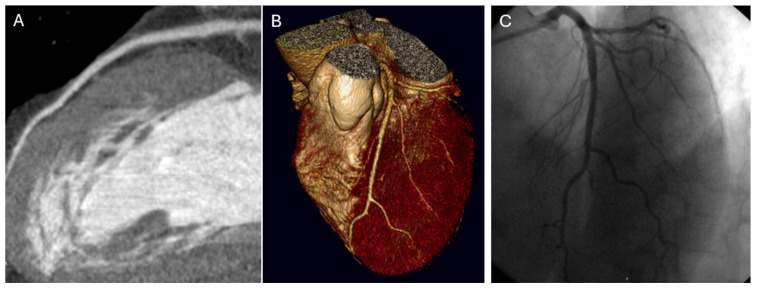
CCTA two-dimensional visualization (**A**) and 3D visualization (**B**) of a patent left anterior descending coronary artery, confirmed by invasive coronary angiography (**C**).

CCTA is a cornerstone non-invasive imaging modality for assessing coronary artery stenosis in patients with suspected coronary artery disease (CAD). Compared with invasive coronary angiography (ICA), CCTA demonstrates high diagnostic accuracy, with reported sensitivities between 85% and 95% and a near-perfect negative predictive value in low- to intermediate-risk populations [32] (Figure 2).

The CONFIRM registry (*n* = 23,854) established that the presence of ≥50% stenosis on CCTA independently predicted all-cause mortality, with greater plaque burden correlating with increased risk [33,34,35,36,37,38]. Importantly, multiple studies have underscored the prognostic significance of identifying non-obstructive coronary plaque. In the ICONIC (Incident Coronary Events Identified by Computed Tomography) study, among 129 culprit lesion precursors identified by CCTA, only 34.6% exhibited ≥50% diameter stenosis and merely 12.8% ≥ 70% stenosis before the occurrence of acute coronary syndrome (ACS) [39]. Similarly, the SCOT-HEART and PROMISE trials demonstrated comparable rates of myocardial infarction between patients with obstructive and non-obstructive CAD [40]. Although additional prospective evidence is warranted, the identification of non-obstructive plaque may have meaningful clinical implications, particularly for guiding preventive therapies such as statins or aspirin.

### 3.1. Assessment of Plaque Burden

CCTA remains the only non-invasive modality capable of quantifying both the extent and the composition of coronary atherosclerosis. Standardized reporting is provided by the CAD-RADS classification, and the updated CAD-RADS 2.0 (2022) further incorporates assessment of stenosis severity, plaque characteristics, and—when available—functional parameters such as CT-derived fractional flow reserve (CT-FFR) or myocardial perfusion imaging [41].

Advances in CCTA technology now allow detailed quantification of calcified and non-calcified plaque components. This can be performed qualitatively (visual scoring), semi-quantitatively, using indices such as the Segment Involvement Score (SIS), Segment Stenosis Score (SSS), or CT-adapted Leaman score, or quantitatively, via semi-automated or automated software tools [41]. Based on attenuation characteristics, plaques are typically categorized as predominantly calcified, predominantly non-calcified, or partially calcified. The term “predominantly” is used intentionally, as histopathologic studies have shown that purely calcified plaques are rare, and non-calcified plaques may contain microcalcifications below the spatial resolution of CCTA [42].

Plaque quantification software typically reports either the absolute volume of atherosclerotic plaque or plaque volume normalized to the corresponding coronary vessel volume—commonly referred to as “plaque burden” or “percentage atheroma volume.” While plaque volume and plaque burden are conceptually similar, burden has the theoretical advantage of accounting for vessel size, thereby adjusting for patient-specific anatomical differences.

Plaque composition is further evaluated using Hounsfield Unit (HU) thresholds: calcified plaque is defined as >350 HU, non-calcified plaque as ≤350 HU, and low-attenuation plaque (LAP) as <30 HU. Because contrast enhancement of the coronary lumen can influence attenuation values, some protocols advocate scan-specific HU thresholds calibrated to proximal luminal attenuation [42].

A recent meta-analysis identified LAP volume and total plaque volume as the most consistently reported plaque features independently associated with MACE. Other characteristics (i.e., plaque volume progression, non-calcified plaque burden, calcified plaque volume, and fibro-fatty plaque volume) were also independently linked to MACE, albeit less consistently. Notably, twelve studies demonstrated that quantitative plaque volume assessment provided incremental prognostic value for MACE prediction compared with traditional surrogate indices such as the CA score, SIS, and CT-adapted Leaman score [43]. Recent evidence further emphasizes the prognostic utility of plaque burden beyond luminal stenosis in patients with established CAD. In a comparative analysis of three markers of atherosclerotic burden (CAC score, degree of stenosis (DS), and SIS) only the CAC score demonstrated a significant association with all-cause mortality. Specifically, CAC scores between 301 and 999 (HR 3.10; 95% CI 1.23–7.80; *p* = 0.017) and ≥1000 (HR 5.81; 95% CI 2.25–15.04; *p* < 0.001) were independent mortality predictors, along with age, smoking, and aspirin use, whereas DS and SIS did not retain prognostic significance in adjusted models. These findings suggest that, in patients with known CAD, a CAC score >300 may serve as a more robust indicator of mortality risk than measures of luminal narrowing or segmental disease extent, supporting its integration into comprehensive plaque assessment and risk stratification [44].

### 3.2. Characterization of Adverse Plaque Features

**Figure 3:**
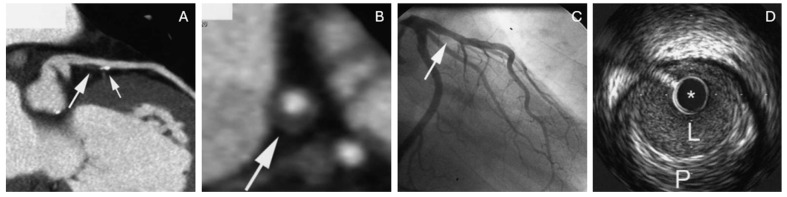
High-risk coronary plaque features: (**A**) Spotty calcification on CCTA. (**B**) Low-attenuation plaque on CCTA. (**C**) Moderate stenosis on invasive coronary angiography. (**D**) IVUS imaging showing the guidewire artifact (*), the residual lumen (L), and plaque burden (P).

Beyond luminal stenosis, CCTA enables detailed evaluation of adverse plaque features (including positive remodeling, low-attenuation core, spotty calcification, and the napkin-ring sign) all of which predict coronary events independently of stenosis severity [23,39,44,45,46,47,48,49,50] (Figure 3). In the SCOT-HEART study, for example LAP burden emerged as the strongest predictor of myocardial infarction, even outperforming CAC and maximal stenosis [23]. Meta-analyses have ranked these features by prognostic impact, identifying the napkin-ring sign as carrying the highest hazard ratio (HR 5.06), followed by LAP (HR 2.95), positive remodelling (HR 2.58), and spotty calcification (HR 2.25), the latter being more prevalent but less specific [48,51,52].

The EMERALD II study provided additional insight into the time-dependent prognostic value of CCTA-derived lesion characteristics in predicting ACS [53]. Among 351 patients who underwent CCTA and subsequently developed ACS within 1 month to 3 years, four lesion parameters were analyzed: stenosis severity, plaque burden, number of high-risk plaque features, and hemodynamic significance by CT-FFR. Lesions associated with early ACS (<1 year) exhibited higher stenosis grades, greater plaque burden, and more pronounced CT-FFR abnormalities, suggesting that morphologically and functionally advanced plaques are more likely to precipitate near-term events. Lesions displaying all four high-risk features had nearly a 50% probability of becoming culprit within two years, while those with three or two features also carried significantly elevated risk compared to baseline (33.0–21.5% vs. 12.1%; *p* < 0.05). These findings highlight the role of CCTA in identifying vulnerable plaques with short- to mid-term ACS potential, reinforcing its clinical value for risk stratification and targeted preventive strategies.

## 4. Fractional Flow Reserve Derived from CT (FFR-CT)

**Figure 4:**
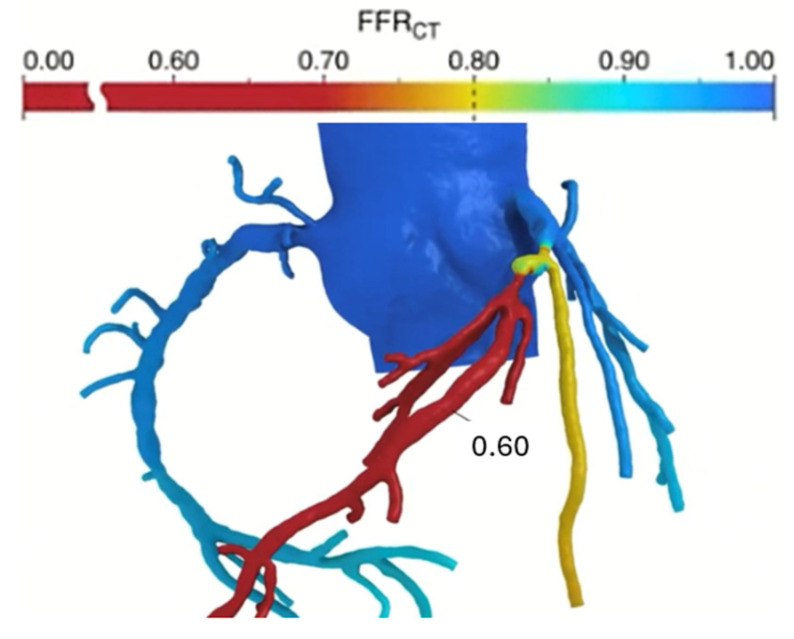
3D FFR-CT visualization showing a critical stenosis of the left anterior descending coronary artery.

FFR-CT represents a major advancement in non-invasive cardiovascular imaging [54,55,56,57,58]. By integrating computational fluid dynamics with standard CCTA datasets, FFR-CT allows for the simultaneous assessment of both coronary anatomy and the physiological significance of coronary stenoses without the need for additional contrast administration, pharmacologic stress, or radiation exposure [22,58,59,60] (Figure 4).

### 4.1. Validation and Diagnostic Performance

The DISCOVER-FLOW trial was among the first pivotal studies to validate the clinical utility of FFR-CT. In 103 patients, it demonstrated a diagnostic accuracy of 84.3%, with sensitivity and specificity of 87.9% and 82.2%, respectively, confirming its functional superiority over anatomical CCTA alone [61]. Subsequently, the DeFACTO trial (*n* = 252) further supported these findings, reporting improved diagnostic discrimination (AUC 0.81 vs. 0.68 for CCTA), with high sensitivity (90%) but modest specificity (54%) [62].

The NXT trial refined the FFR-CT methodology by enforcing rigorous image quality criteria and introducing updated computational algorithms. These refinements improved diagnostic accuracy (81%), sensitivity (86%), and specificity (79%), underscoring the critical role of image quality and algorithmic precision in optimizing FFR-CT performance [63]. A 2019 meta-analysis encompassing 24 studies confirmed the robust diagnostic efficacy of FFR-CT, reporting superior accuracy with on-site computation (84.1%) compared to off-site analysis (79.3%), and highlighting the relevance of workflow optimization [32].

### 4.2. Clinical Utility and Real-World Evidence

Real-world studies have reinforced the clinical and practical value of FFR-CT. The PLATFORM study demonstrated a 61% reduction in unnecessary invasive coronary angiography, validating FFR-CT as an effective gatekeeper in the diagnostic pathway [33]. Similarly, the ADVANCE registry, including over 5000 patients, established the safety of deferring angiography in patients with FFR-CT values >0.80, with low adverse event rates under conservative management [31].

The SYNTAX III Revolution trial extended these findings to multivessel CAD, reporting a strong concordance between FFR-CT-guided and angiography-guided revascularization strategies (κ = 0.82), and demonstrating that FFR-CT assessment led to changes in revascularization planning in 16% of cases [33]. In the FASTTRACK CABG trial, surgical planning guided exclusively by FFR-CT accurately identified ischemic territories and allowed functional derivation of SYNTAX scores [35]. The PACIFIC and FORECAST trials further corroborated the diagnostic value of FFR-CT, showing comparable accuracy to cardiac PET and reinforcing its potential as a comprehensive, non-invasive alternative for ischemia assessment [36].

Despite its strong diagnostic and clinical performance, FFR-CT has several limitations. High-quality CCTA acquisition is essential, as artifacts from motion, calcification, or suboptimal contrast may render up to 25% of studies non-diagnostic [64,65]. The technique relies on population-averaged assumptions for hyperaemic flow, which may not adequately reflect patient-specific hemodynamic—particularly in cases of microvascular [66] dysfunction or post-STEMI, where diagnostic accuracy can decrease to approximately 70% [67,68].

Further challenges include methodological heterogeneity across studies (scanner technology, population characteristics, and selection bias) that limit generalizability. Intermediate FFR-CT values (0.75–0.80) often necessitate additional functional testing. Cost and accessibility remain relevant barriers, especially when using cloud-based platforms that depend on proprietary software and off-site data processing.

Nonetheless, technological advances are promising. Recent evidence indicates that artificial intelligence–based FFR-CT platforms can achieve diagnostic accuracies approaching 95%, with processing times under 10 min [69,70]. The introduction of photon-counting CT technology is also expected to enhance spatial resolution, mitigate artifacts, and improve diagnostic accuracy even in patients with coronary stents or extensive calcifications [71].

On-site FFR-CT analysis represents another promising frontier. Meta-analyses report higher diagnostic accuracy, sensitivity, and specificity for on-site implementations (84.1%, 83.4%, and 84.7%, respectively) compared with off-site systems [22]. Collectively, these developments highlight the evolving role of FFR-CT as a bridge between anatomical and functional imaging, with growing potential for integration into routine clinical practice and personalized decision-making in CAD management.

## 5. Coronary CT Angiography in Chronic Coronary Syndromes

In patients with chronic coronary syndromes (CCS), the balance between anatomical and functional testing for the evaluation of coronary artery disease (CAD) has been a longstanding topic of discussion [72,73]. The 2024 ESC Guidelines on CCS place renewed emphasis on anatomical imaging with CCTA as the preferred first-line modality over functional testing, largely supported by evidence from recent clinical trials [74].

The PROMISE trial compared an initial diagnostic strategy based on functional testing, such as stress echocardiography or nuclear perfusion imaging, with CCTA in patients presenting with stable chest pain. Over a median follow-up of 25 months, cardiovascular outcomes were similar between the two groups, indicating clinical equipoise between functional and anatomical approaches [75]. In contrast, the SCOT-HEART trial demonstrated that incorporating CCTA into standard care resulted in a significant reduction in major adverse cardiovascular events (MACE) over a 4.8-year follow-up period, underscoring its prognostic value. Hence, the choice between anatomical and functional testing should be individualized, taking into account the patient’s clinical presentation, cardiovascular risk profile, and local availability of advanced imaging modalities. The use of CCTA should be guided by the pre-test likelihood of CAD, as estimated using validated models such as the RF-CL tool, which integrates symptoms, risk factors, and demographic variables [76].

For patients with suspected chronic coronary syndromes and low to moderate pre-test likelihood of obstructive CAD (5–50%), CCTA is recommended as the first-line diagnostic modality. It provides a comprehensive evaluation of coronary anatomy, facilitates risk stratification for MACE, and refines diagnosis in cases where other non-invasive tests yield inconclusive results [77,78].

## 6. Coronary CT Angiography in the Acute Setting

In the acute setting, CCTA plays an increasingly important role in the diagnostic evaluation of patients presenting with suspected acute coronary syndrome (ACS). According to the 2023 ESC Guidelines for ACS, CCTA should be considered in selected patients with suspected ACS who have non-elevated or uncertain troponin levels, non-diagnostic ECGs, and no recurrent symptoms [78,79].

CCTA demonstrates a high negative predictive value (NPV) of approximately 90.9%, as reported by Linde et al., allowing rapid and reliable exclusion of ACS and aiding in the identification of alternative life-threatening conditions such as pulmonary embolism or aortic dissection [80]. In a sub-analysis of the VERDICT trial, CCTA maintained a high positive predictive value (89.9%) for detecting obstructive CAD, even when non-diagnostic scans were included [81].

Moreover, Kofoed et al. confirmed the long-term prognostic equivalence between CCTA and invasive coronary angiography (ICA) in ACS management. Rates of composite endpoints—including all-cause death, recurrent myocardial infarction, ischemia-related hospitalization, and heart failure—were similar regardless of whether CAD was identified by CCTA or ICA [82]. Collectively, these findings underscore the expanding role of CCTA as a rapid, accurate, and non-invasive diagnostic tool in both chronic and acute coronary settings, bridging the gap between anatomical definition and clinical decision-making.

## 7. Perivascular Fat Attenuation Index (pFAI) Assessment

**Figure 5:**
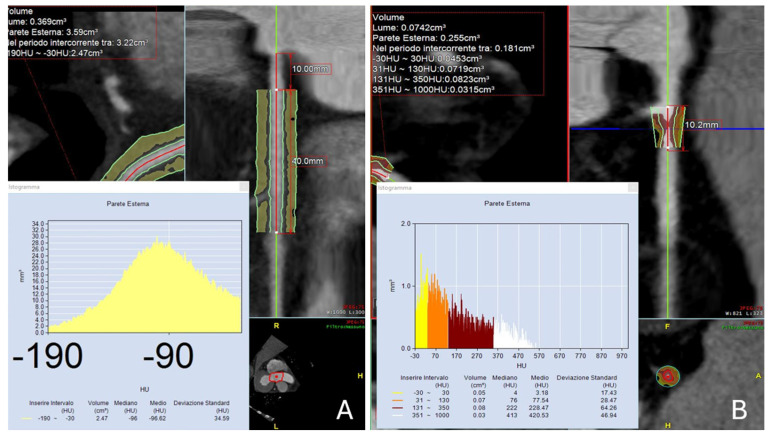
pFAI evaluation on CT analysis. (**A**) Plaque in the right coronary artery showing normal pFAI. (**B**) High-risk coronary plaque in the left anterior descending artery showing elevated pFAI.

The Perivascular Fat Attenuation Index (pFAI) is an emerging CCTA-derived biomarker that quantifies inflammatory activity within the pericoronary adipose tissue (PCAT) [83,84,85,86,87]. By analyzing spatial gradients in PCAT attenuation, pFAI indirectly captures inflammatory changes occurring in adjacent coronary vessels [88,89]. This association stems from inflammation-induced alterations in PCAT phenotype—such as modifications in adipocyte size, lipid composition, and differentiation—that reflect underlying vascular inflammatory states [90] (Figure 5). Recent advancements in image-processing algorithms have enabled reproducible and quantitative evaluation of pFAI gradients across different coronary territories using dedicated software platforms.

### 7.1. Prognostic Role of pFAI

The prognostic significance of pFAI was first established in the CRISP-CT study, a large multicenter post hoc analysis, which showed that elevated pFAI values (≥–70.1 HU) in the proximal right coronary artery (RCA) and left anterior descending (LAD) (but not the left circumflex) were independent predictors of both all-cause and cardiac mortality [91].

Similarly, Kuneman et al. reported higher pFAI values around precursor segments of culprit lesions compared to stable or non-culprit plaques in patients with suspected CAD, reinforcing the link between vascular inflammation, plaque vulnerability, and lesion instability [92].

The clinical implementation of this biomarker has been facilitated by CaRi-Heart^®^, a CE-marked software platform that computes artery-specific FAI-Scores adjusted for technical (e.g., tube voltage), anatomical, and demographic factors [93]. The system also generates a CaRi-Heart^®^ Risk, an individualized 8-year risk estimate for fatal cardiac events that integrates pFAI-derived inflammation data with traditional cardiovascular risk factors and CT-based indices of plaque burden (e.g., Duke CAD Index).

This approach exemplifies the ongoing transition of CCTA from a purely anatomical imaging tool toward a comprehensive risk stratification modality incorporating inflammatory phenotyping.

### 7.2. Inflammation and Residual Risk

The ORPHAN study provided population-level evidence for the incremental value of pFAI in cardiovascular risk prediction [94]. Among 40,091 patients undergoing clinically indicated CCTA, only one-third of MACE occurred in those with obstructive CAD. Notably, approximately 25% of patients without obstructive lesions exhibited elevated inflammatory risk, reflected by abnormal pFAI values, and experienced a tenfold increase in cardiac mortality or MACE over a 10-year period.

A dose–response relationship was observed between the number of coronary arteries with elevated pFAI and adverse outcomes, underscoring the additive prognostic impact of systemic coronary inflammation. These findings advocate for integrating pFAI into standard CCTA-based risk assessment, particularly in patients with non-obstructive CAD who may otherwise be under-recognized as high risk [95].

### 7.3. Integration with Plaque Phenotyping and Therapy

Naniwa et al. recently provided compelling evidence for the additive prognostic value of coronary inflammation and high-risk plaque characteristics, as assessed by CCTA in patients undergoing percutaneous coronary intervention (PCI) [96]. The study demonstrated that combining conventional cardiovascular risk factors with CT-derived high-risk plaque features significantly improved prediction of the composite endpoint—cardiovascular death, non-fatal myocardial infarction, revascularization, and stroke. Importantly, the inclusion of PCAT-based inflammation metrics further enhanced model performance, achieving an area under the curve (AUC) exceeding 0.80. Patients with elevated coronary inflammation exhibited markedly higher rates of target vessel and lesion failure after PCI, including increased repeat revascularizations [96]. These associations persisted even after adjustment for plaque burden and established risk factors, highlighting the independent prognostic contribution of residual inflammatory risk.

Crucially, the study also identified a therapeutic interaction between inflammation and statin efficacy: patients with high coronary inflammation derived significant benefit from statin therapy (HR 0.46, 95% CI: 0.24–0.88), whereas those with low inflammation did not (HR 0.94, 95% CI: 0.19–4.61). These findings reinforce the emerging paradigm of inflammation-guided statin therapy and align with recent evidence suggesting that targeting coronary inflammation may reduce lifetime MACE by approximately 30% [97]. Collectively, these data support the integration of plaque morphology, inflammation biomarkers, and personalized therapy selection into the pre-PCI assessment workflow, potentially informing precision strategies for secondary prevention [84].

## 8. Stress-CTP

The impetus for adopting stress computed-tomographic myocardial perfusion (stress-CTP) stems from the well-recognized limitation of purely anatomical imaging with coronary CT angiography (CCTA) in reliably determining the functional significance of coronary stenoses. While CCTA excels in the morphological visualization of coronary anatomy, it may overestimate the haemodynamic relevance of lesions, particularly in the presence of heavy calcification, diffuse disease, or prior revascularisation. To overcome this limitation, stress-CTP was developed as a hybrid strategy that merges anatomical and functional assessment in a single session, thereby providing both vessel morphology and myocardial perfusion under pharmacologic stress. Dynamic myocardial perfusion CT allows quantification of myocardial blood flow (MBF) during vasodilator administration, enabling the identification of territories with impaired perfusion despite non-severe stenoses—or conversely, ruling out ischemia in anatomically significant but functionally benign lesions [97]. The stress-CTP workflow follows a structured sequence designed to integrate anatomical and functional cardiac assessment within a single imaging session. The examination typically comprises three key phases: pre-scan preparation, stress perfusion acquisition, and rest CCTA [98,99,100] (Table 2). Radiation exposure for combined CCTA + stress CTP is typically 6–9 mSv with iterative or deep-learning reconstruction, markedly lower than early studies [98]. The entire protocol—from patient preparation to reconstruction—can be completed in under 30 min, providing a comprehensive anatomical and functional cardiac evaluation in a single, efficient examination.

The clinical purpose of stress CTP is multifold: (1) to identify hemodynamically significant coronary stenoses warranting revascularization or intensification of medical therapy; (2) to provide prognostic information by quantifying impaired perfusion and microvascular dysfunction; (3) to streamline diagnostic pathways by combining anatomical and functional assessment in a “one-stop” scan rather than separate CCTA plus stress testing or PET/CMR; and (4) to optimize patient selection for invasive angiography, thereby reducing unnecessary procedures.

Prospective studies have demonstrated that combining CCTA with stress CTP improves diagnostic accuracy (AUC ≈ 0.919) compared to CCTA alone (AUC ≈ 0.826). Moreover, parameters such as the Stress Flow Ratio (SFR), derived from dynamic CTP, have shown excellent specificity (≈91%) for detecting flow-limiting lesions when added to CCTA stenosis assessment [101]. Nevertheless, the protocol requires high-end, wide-coverage CT scanners, precise synchronization of contrast delivery and stress timing, and meticulous radiation dose management. Quantitative flow values are not yet fully standardized across vendors, and larger multicentre outcome studies are still needed to define robust diagnostic thresholds and prognostic cut-offs.

## 9. Current Limitations and Practical Considerations

Cardiac CT has evolved into a powerful multiparametric tool for cardiovascular assessment; however, several limitations should be considered when interpreting its clinical application. First, even if radiation exposure has been significantly reduced with modern acquisition strategies, it may still represent a relevant factor in selected patient groups or repeated examinations. Access to advanced CT technologies and post-processing tools is not uniform across institutions, and cost-related issues may influence broader implementation, particularly for emerging techniques such as CT-derived functional and tissue characterization biomarkers. In addition, variability in acquisition protocols, post-processing approaches, and vendor-specific software solutions may affect the reproducibility of quantitative parameters, highlighting the need for further standardization across centers and imaging platforms. Cost-effectiveness and accessibility also remain important considerations for the widespread adoption of advanced applications such as AI-based FFR-CT, stress-CTP, and pFAI. Furthermore, several CT-derived biomarkers and AI-driven tools remain in the validation phase and are not yet fully integrated into routine clinical workflows. To place cardiac CT within the broader diagnostic pathway for coronary artery disease, a comparative overview with other commonly used imaging modalities is provided in Table 3. Overall, no single imaging modality is universally superior, and their use should be tailored to clinical context, patient characteristics, and local expertise. Cardiac CT plays a central role particularly in the non-invasive exclusion of coronary artery disease, while functional and invasive techniques remain complementary in specific clinical scenarios.

Future developments in cardiac CT are expected to further expand its clinical utility. Advances in scanner technology, including improved temporal and spatial resolution and dose reduction strategies, are likely to enhance image quality and broaden applicability to more complex patient populations. In parallel, the integration of artificial intelligence for image reconstruction, plaque characterization, and automated functional assessment may improve diagnostic accuracy and workflow efficiency. Emerging techniques such as photon-counting CT and more refined computational models for functional assessment also hold promise for further strengthening the role of cardiac CT in comprehensive cardiovascular evaluation. Nevertheless, further prospective multicenter studies are needed to validate the reproducibility, prognostic value, and clinical impact of emerging techniques such as pFAI and stress-CTP across different patient populations, scanner technologies, and clinical settings before their widespread implementation can be fully established.

## 10. Conclusions

Cardiac CT has transitioned from an anatomical imaging modality to a comprehensive framework for cardiovascular precision medicine. CAC scoring refines primary and secondary prevention strategies; CCTA, enhanced by FFR-CT and stress CTP, delivers accurate anatomical–functional characterization of coronary disease; and pFAI provides a novel window into vascular inflammation and residual risk.

Together, these complementary techniques enable a multidimensional evaluation of coronary health (structural, functional, and biological) informing tailored preventive and therapeutic strategies. The convergence of artificial intelligence and next-generation CT technologies further enhances diagnostic precision, workflow efficiency, and prognostic value (Table 4).

As cardiac CT continues to integrate quantitative plaque metrics, inflammation imaging, and clinical decision algorithms, it is poised to become the central non-invasive tool for holistic cardiovascular phenotyping. In this context, its evolution mirrors the broader trajectory of modern cardiology from detecting disease to anticipating it.

## Figures and Tables

**Table 1:** Coronary Artery Calcium (CAC) Score: Clinical Interpretation and Management Implications.

CAC Score (AU)	Plaque Burden	10-Year Risk Estimate	Clinical Implication	Recommended Action
0	No calcified plaque	Very low (<1%)	Very low CHD/CVD event rate (“warranty period” 5–10 years)	May defer statin therapy; reinforce lifestyle; consider retesting in 5–10 years
1–10	Minimal	Low	Early atherosclerosis possible; risk slightly higher than 0	Lifestyle modifications; consider statins if ≥1 major risk factor
11–99	Mild	Moderate (1–10%)	Evidence of coronary atherosclerosis	Statin therapy generally recommended, especially in intermediate-risk patients
101–299	Moderate	Intermediate (10–20%)	Substantial atherosclerosis; increasing event risk	Statin indicated; intensify preventive strategies; consider aspirin in select cases
300–999	Severe	High (>20%)	Extensive atherosclerosis; high CHD event rate	Statin + aspirin likely indicated; aggressive risk factor management
≥1000	Very severe	Very high (>25–30%)	Comparable to secondary prevention risk; mortality elevated	Maximal intensity statins; full secondary prevention strategy justified

**Table 2:** Workflow of Stress-CTP: acquisition phases, technical parameters, and clinical objectives.

Phase	Key Steps	Technical Details/Parameters	Purpose/Notes
1. Pre-scan preparation	Patient preparation and monitoring	Fasting ≥ 3–4 h Avoid caffeine ≥ 24 h Beta-blockers for HR < 65 bpm IV access (18–20 G) ECG gating setup	Optimize physiological conditions and minimize heart rate variability; prevent artifacts; ensure vasodilator safety
2. Stress induction	Pharmacologic hyperemia	Adenosine 140 µg/kg/min for 3–4 min or Regadenoson 0.4 mg bolus	Achieve maximal coronary vasodilation for perfusion assessment
3. Contrast injection	First-pass contrast delivery	Iodinated contrast 50–70 mL (5–6 mL/s) + 40 mL saline flush Injection triggered at peak hyperemia	Provide opacification of myocardial microcirculation during stress
4. Stress CT perfusion acquisition	Dynamic or static imaging	Dynamic CTP: 8–12 sequential low-dose phases over ≈20–30 s Static CTP: single acquisition at peak enhancement Coverage: ≥256 slices (≥14 cm *z*-axis) ECG-gated, tube voltage 80–100 kVp	Measure absolute MBF or identify hypoattenuated perfusion defects; whole-heart coverage avoids shuttle artifacts
5. Rest CCTA	Coronary anatomy imaging	Performed 10–15 min after stress phase ECG-gated helical or prospective mode Contrast 50–70 mL Iterative or DLIR reconstruction	Evaluate coronary anatomy, stenosis severity, and plaque morphology; enable comparison of stress vs. rest
6. Image post-processing	Quantitative and qualitative analysis	Generate MBF (mL/min/100 g) and MFR (stress/rest ratio) Color-coded perfusion maps co-registered with coronary tree Semi-automated software analysis	Integrate anatomical and perfusion data for comprehensive functional CAD assessment
7. Radiation and total time	Dose optimization and workflow	Typical combined dose 6–9 mSv (CTP + CCTA) Exam duration ≈ 25–30 min Iterative/DL reconstruction to minimize dose	Achieve full anatomical–functional evaluation with acceptable radiation exposure and short acquisition time

CTP, Computed Tomography Perfusion; CCTA, Coronary Computed Tomography Angiography; MBF, Myocardial Blood Flow; MFR, Myocardial Flow Reserve; HR, Heart Rate; kVp, Kilovolt Peak; DLIR, Deep Learning Image Reconstruction.

**Table 3:** Comparative overview of cardiac CT and alternative imaging modalities for the evaluation of coronary artery disease.

Modality	Primary Clinical Role	Diagnostic Performance (CAD)	Strengths	Limitations	Radiation Exposure
CCTA	Anatomic assessment of coronary arteries; rule-out CAD	High sensitivity, high negative predictive value; moderate specificity (improves with FFR-CT when available)	Excellent rule-out test; non-invasive coronary visualization; fast acquisition; prognostic plaque characterization	Limited by heavy calcifications, high/irregular HR (partially mitigated by modern scanners); contrast use; incidental findings	Low–moderate (depending on protocol, often ~1–5 mSv in contemporary protocols)
Stress echocardiography	Functional ischemia detection	Moderate sensitivity and specificity; operator-dependent	Widely available; no radiation; bedside; low cost; real-time functional assessment	Image quality dependent on acoustic window; limited coronary anatomy assessment; operator variability	None
MRI (stress perfusion/viability)	Myocardial ischemia, viability, tissue characterization	High diagnostic accuracy for ischemia and scar	No ionizing radiation; excellent tissue characterization; gold standard for volumes/function	Limited availability; longer acquisition; contraindications (devices, claustrophobia); expertise required	None
PET	Quantitative myocardial perfusion and ischemia	Very high sensitivity; high diagnostic accuracy; strong prognostic value	Quantitative flow assessment (MBF, CFR); excellent accuracy in multivessel disease	High cost; limited availability; radiotracer logistics; radiation exposure	Moderate
ICA	Gold standard for coronary lumen assessment; allows intervention	Very high spatial resolution for lumen stenosis	Allows immediate revascularization (PCI); highest spatial resolution	Invasive; does not assess plaque composition well; procedural risk; overestimation of functional significance if not combined with FFR	Moderate

CCTA, coronary computed tomography angiography; CAD, coronary artery disease; PET, positron emission tomography; CMR, cardiac magnetic resonance; ICA, invasive coronary angiography; MBF, myocardial blood flow; CFR, coronary flow reserve; FFR-CT, fractional flow reserve derived from coronary CT angiography.

**Table 4:** Clinical role and evidence level of CT-derived coronary imaging biomarkers across established and emerging applications.

Method	Clinical Status	Evidence Level	Main Clinical Use	Comments
CAC score	Established	High	Cardiovascular risk stratification in asymptomatic and intermediate-risk patients	Supported by large cohort studies and guideline recommendations
Stenosis assessment	Established	High	Evaluation of coronary artery disease in patients with stable chest pain	Recommended in current ESC guidelines for CAD evaluation
Plaque burden assessment	Established/Adjunctive	Moderate–High	Additional risk refinement beyond luminal stenosis	Improves prognostic stratification compared to stenosis alone
High-risk plaque features	Emerging	Moderate	Identification of vulnerable plaque phenotype	Incremental prognostic value, limited standardization across studies
Perivascular adipose tissue attenuation	Emerging	Moderate	Assessment of coronary inflammation	Promising prognostic marker; ongoing validation in outcome studies
CT-FFR	Emerging	Moderate–High	Functional assessment of lesion-specific ischemia	Increasing clinical adoption, but limited availability
Radiomics/AI-based CT biomarkers	Investigational	Low–Moderate	Risk prediction and phenotyping of CAD	Currently research-focused; requires external validation

CAC, Coronary Artery Calcium; CT, Computed Tomography; CT-FFR, Computed Tomography–derived Fractional Flow Reserve; CAD, Coronary Artery Disease; AI, Artificial Intelligence.

## Data Availability

All data is contained within the article.

## Abbreviations

The following abbreviations are used in this manuscript:

ACS	acute coronary syndrome
CAC	coronary artery calcium
CAD	coronary artery disease
CAD-RADS	coronary artery disease reporting and data system
CCTA	Coronary CT Angiography
CCS	chronic coronary syndrome
CVD	cardiovascular disease
CT	Computed tomography
CT-FFR	CT fractional flow reserve
CTP	CT perfusion
ECG	electrocardiogram
ESC	European Society of Cardiology
HU	Hounsfield units
ICA	invasive coronary angiography
MACE	major adverse cardiovascular events
MESA	Multi-Ethnic Study of Atherosclerosis
NNT10y	Number need to treat for 10 years
PCAT	pericoronary adipose tissue
PFAI	perivascular fat attenuation index
RF-CL	Risk Factor-weighted Clinical Likelihood
SCORE2	systematic coronary risk estimation 2
SCOT-HEART	Scottish Computed Tomography of the HEART
